# Supplementary material for: Open syntaxin overcomes exocytosis defects of diverse mutants in C. elegans
Source: Nat Commun. 2020 Nov 2;11:5516. doi: 10.1038/s41467-020-19178-x (PMC7606450; doi:10.1038/s41467-020-19178-x)
Supplement: Supplementary file 1 — Supplementary Information [file 41467_2020_19178_MOESM1_ESM.pdf]

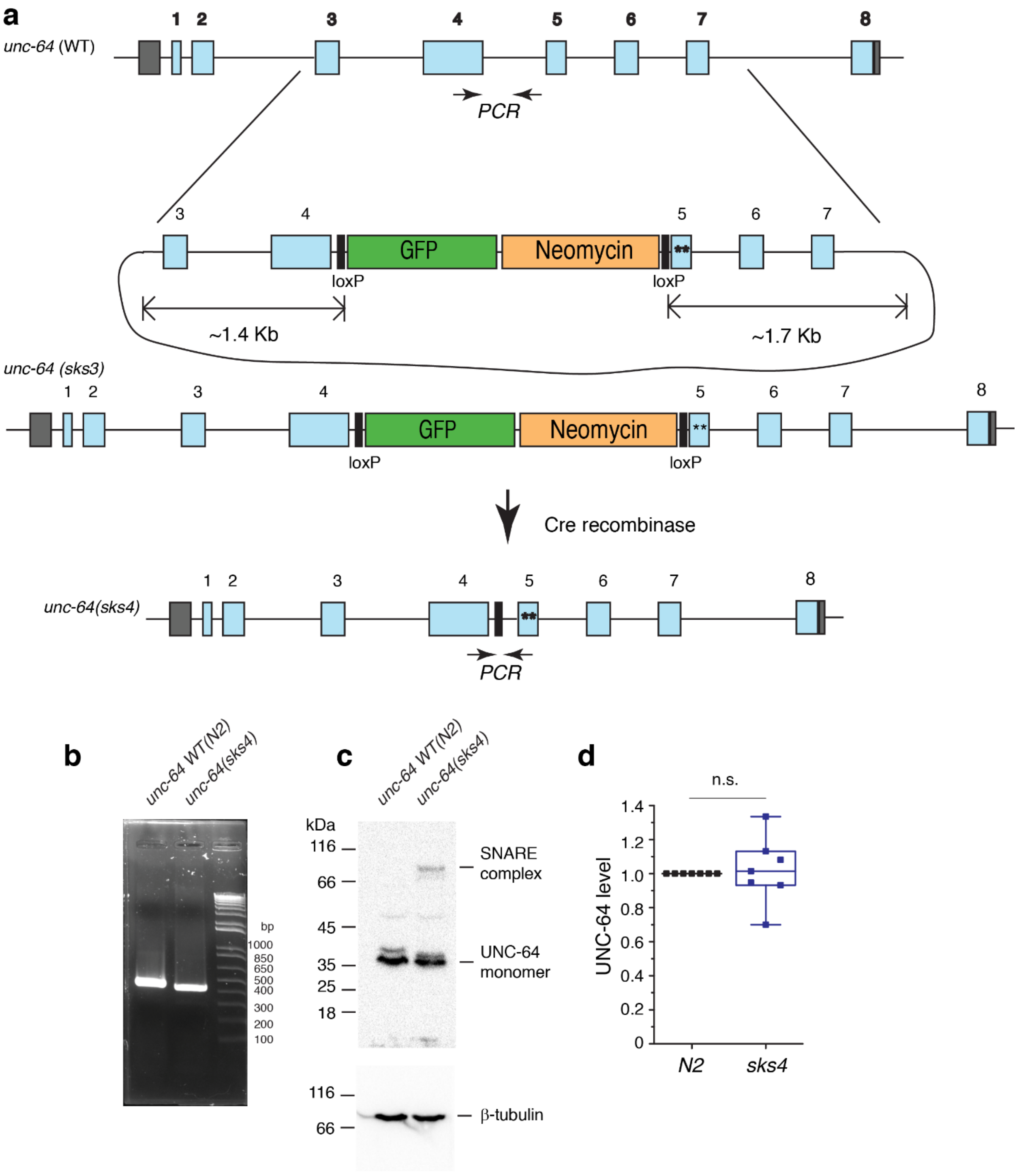

Supplementary Figure S1. Generation of open syntaxin KI animals. (a) A schematic diagram represents how CRISPR-mediated homologous recombination replaces the wild-type allele of *unc-64* with the L166A/E167A mutant allele. (b) PCR-based genotyping of *unc-64(sks4)* LE open mutant allele in comparison with WT. The results were confirmed three times independently. (c) Fifty micrograms of indicated samples were subjected to SDS-PAGE followed by immunoblotting. The expression of UNC-64 and β-tubulin (loading control) proteins in the blot was serially detected with rabbit anti-syntaxin-1 polyclonal antibody (I378) and mouse anti-β-tubulin monoclonal antibody (E7), respectively, followed by enhanced chemiluminescence detection. We provide uncropped and

unprocessed scans in the Source Data file. We prepared 3 independent SDS homogenate samples from WT and open syntaxin animals and prepared 7 blots from these samples. (d) Box-and-whisker plots overlaid with data points show that there was no significant difference between N2 WT UNC-64 and open UNC-64(sks4) signals, which were normalized by β-tubulin signal. n.s. = not significant, n=7, two-sided paired-t-test, $t_{(6)}=-0.268$, $p = 0.798$. Box-and-whisker plots represent the median (central line), 25th–75th percentile (bounds of the box) and 5th–95th percentile (whiskers).

WT:...tctagattttggagcacacctgaatcatggtgctcccaatgt*ctgctacctgcctac*
***sks4*:.tctagattttggagcacacctgaatcatggtgctcccaatgtctgctacctgccgcg**

*aagg*cggcttacgcctacgacatatatgtatacctgtcatagcgc*aagacgaacccagagaac*
gccgcagtggtcctgcaactataacttcgtataatgtatgctatacgaagttataggccgcct

*at*cggacgcttcaaaaaaatttttgtcaggcacaccgactagtcgaattataagtatcgtagg
gatg-------------------------------cgactagtcgaattataagtatcgtagg

caggtaggcaggcagacgtgtaggcctaactggggcaggacaaaatttttcctgacattctgt
caggtaggcaggcagacgtgtaggcctaactggggcaggacaaaatttttcctgacattctgt

gccactaaattttggatcagtttttaaaaaaattcacacaaaattctaataatccattacata
gccactaaattttggatcagtttttaaaaaaattcacacaaaattctaataatccattacata

A G K Q V G D E D **L/AE/A** *E M I E S G N*
ttttttcaGCTGGAAAACAAGTCGGAGATGAGGATTTGGAGGAAATGATTGAGAGCGGAAATC
ttttttcaGCTGGAAAACAAGTCGGAGATGAGGATGCTGCAGAAATGATTGAGAGCGGAAATC

P *G V F T Q G I I T D*
CGGGAGTATTTACACAAGGAATCATCACAGATAC
CGGGAGTATTTACACAAGGAATCATCACAGATAC

Supplementary Figure S2. Comparison of genomic *unc-64* gene (WT allele from N2 vs. *sks4* allele) surrounding the L166A/E167A mutations (highlighted by red). Lower letters indicate intron while capital letters indicate exon of *unc-64* genome. Different sequences between the two in the intron region were highlighted by yellow. Blue letters indicate loxP sequence. Underlines indicated the target sequences of Cas9.

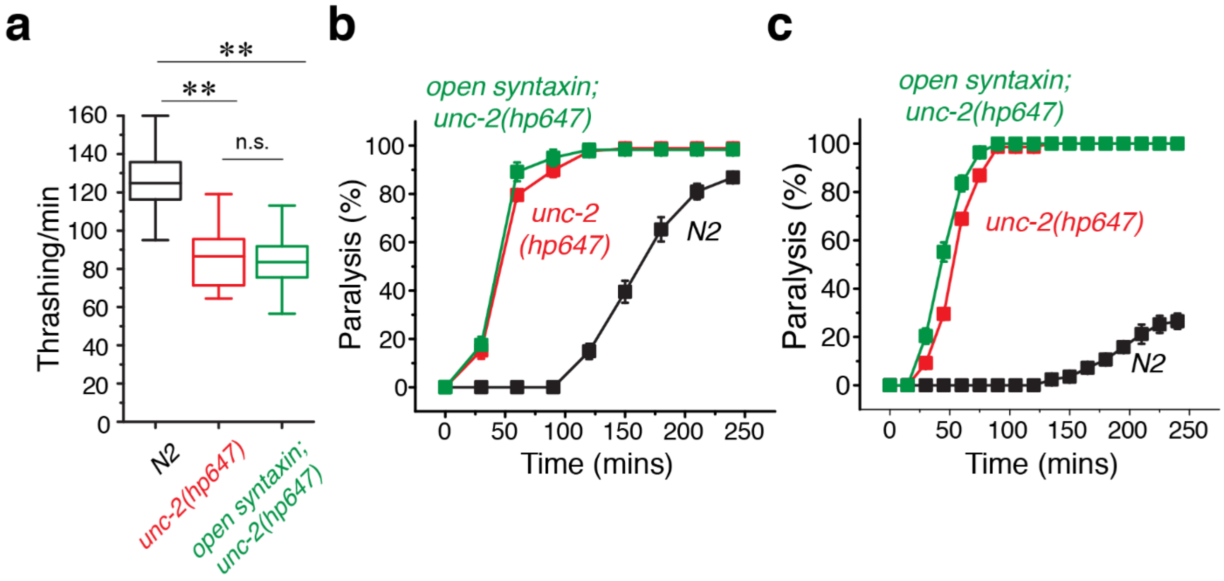

Supplementary Figure S3. Open syntaxin enhances exocytosis in gain-of-function calcium channel mutants. (a) Box-and-whisker plots of thrashing assay for N2, *unc-2(hp647)*, and *open syntaxin; unc-2(hp647)* double mutants in M9 buffer. Interestingly, *unc-2(hp647)* worms displayed reduced thrashing rates (84.7 thrashes/min) compared to N2 wild-types, which was unaffected by the introduction of open syntaxin KI in the double mutant (85.2 thrashes/min). n=40 for each strain. In one-way ANOVA statistical tests, $F_{(2,117)}$ = 92.5 and p = 0.00. Tukey's test was performed for means analysis in ANOVA. For N2 vs unc-2(hp647): ** p = 0.00; For *unc-2(hp647)* vs. *open syntaxin; unc-55(hp647)*: n.s. p = 0.99; For N2 vs *open syntaxin; unc-2(hp647)*: ** p = 0.000. Box-and-whisker plots represent the median (central line), 25th–75th percentile (bounds of the box) and 5th–95th percentile (whiskers). (b) Aldicarb assays of N2, *unc-2(hp647)*, and *open syntaxin; unc-2(hp647)* double mutants. *unc-2(hp647)* and *open syntaxin; unc-2(hp647)* animals displayed similar hypersensitivity to 1 mM aldicarb. n=6. Each assay was conducted with 15-20 worms. Error bars represent SEM. (c) Aldicarb assays of N2, *unc-2(hp647),* and *open syntaxin; unc-2(hp647)* double mutants on 0.3 mM aldicarb plates and probed for paralysis every 15 mins. The *open syntaxin; unc-2(hp647)* animals displayed slightly greater hypersensitivity to aldicarb. n=6. Each assay was conducted with 15-20 worms. Error bars represent SEM.

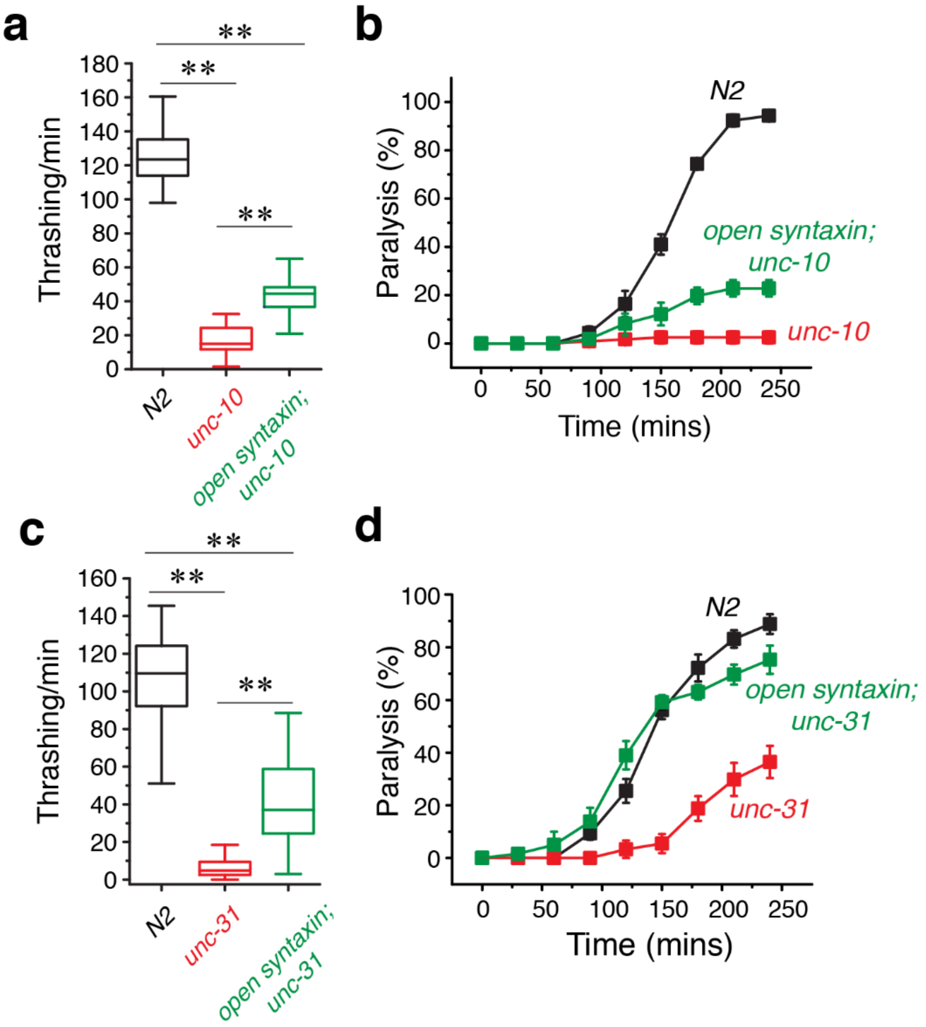

Supplementary Figure S4. Open syntaxin partially rescues exocytosis and motility of *unc-10* and *unc-31* mutants. (a) Box-and-whisker plots of thrashing assay for N2, *unc-10(md1117)*, and *open syntaxin; unc-10(md1117)* double mutants in M9 buffer. *unc-10(md1117)* worms displayed greatly reduced thrashing rates (17.3 thrashes/min) which was moderately increased by the introduction of open syntaxin in the double mutant (42.5 thrashes/min). n=40 for each strain. In one-way ANOVA statistical tests, $F_{(2,117)}$ = 726 and p = 0.00. Tukey's test was performed for means analysis in ANOVA. For all comparisons, ** p = 0.00. (b) Aldicarb assays of N2, *unc-10*, and *open syntaxin; unc-10(md1117)* double mutants. *open syntaxin; unc-10(md1117)* animals displayed slightly increased sensitivity to aldicarb compared to *unc-10(md1117)* single mutants. n=6. Each assay was conducted with 15-20 worms. Error bars represent SEM. (c) Box-and-whisker plots of thrashing assay for N2, *unc-31(e928)*, and *open syntaxin; unc-31(e928)* double mutants in M9 buffer. *unc-31(e928)* worms displayed impaired thrashing rates (8.31 thrashes/min) which was increased by the introduction of open syntaxin in the double mutant (39.6 thrashes/min). n=40 for each strain. In one-way ANOVA statistical tests, $F_{(2,117)}$ = 281 and p = 0.00. Tukey's test was performed for means analysis in ANOVA. For all comparison, ** p = 0.00. (d) Aldicarb assays of N2, *unc-31(e928)*, and *open syntaxin; unc-31(e928)* double mutants. *open syntaxin; unc-31(e928)* animals displayed increased sensitivity to aldicarb to near wild-type levels compared to *unc-31(e928)* single mutants. n=6. Each assay was conducted with 15-20 worms. Error bars represent SEM. Box-and-whisker plots all represent the median (central line), 25th–75th percentile (bounds of the box) and 5th–95th percentile (whiskers).

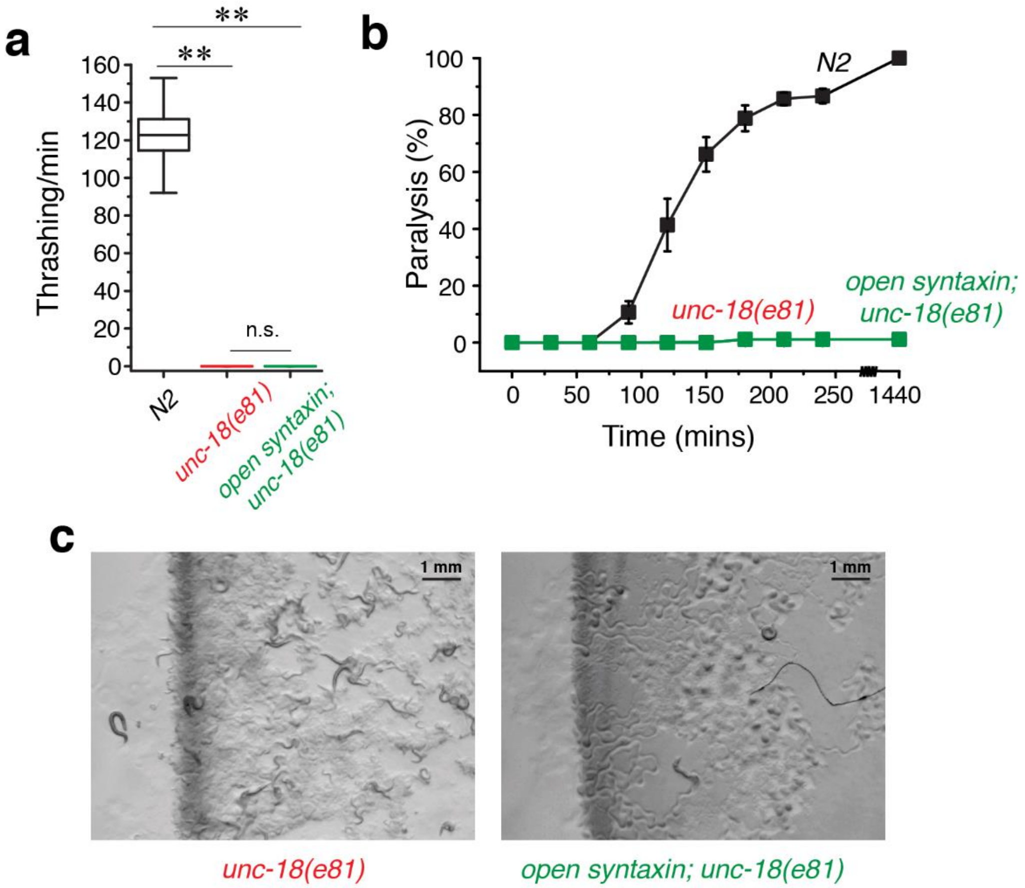

Supplementary Figure S5. The knock-in open syntaxin mutation further impairs phenotypes of *unc-18(e81)* mutants. (a) Box-and-whisker plots of thrashing assay of N2, *unc-18(e81),* and *open syntaxin; unc-18(e81)* double mutants in M9 buffer. *unc-18(e81)* worms displayed greatly reduced thrashing rates (0.200 thrashes/min) which was reduced further by the introduction of open syntaxin in the double mutant to 0.100 thrashes/min. n=40 for each strain. In one-way ANOVA statistical tests, $F_{(2,117)}$ = 2170 and p = 0.00. Tukey's test was performed for means analysis in ANOVA. For N2 vs *unc-18(e81)*: ** p = 0.00; for *unc-18(e81)* vs. *open syntaxin; unc-18(e81)*: n.s. p = 1.00; for N2 vs. *open syntaxin; unc-18(e81)*: ** p = 0.00. Box-and-whisker plots represent the median (central line), 25th–75th percentile (bounds of the box) and 5th–95th percentile (whiskers). (b) Aldicarb assays of N2, *unc-18(e81),* and *open syntaxin; unc-18(e81)* double mutants. *unc-18* and *open syntaxin; unc-18* animals displayed similar resistance to aldicarb even after 24 hours. n=6 for 4-hr assays, n=1 for 24-hr assay. Each assay was conducted with 15-20 worms. Error bars represent SEM. (c) Growth rate/body size images taken at day 9.

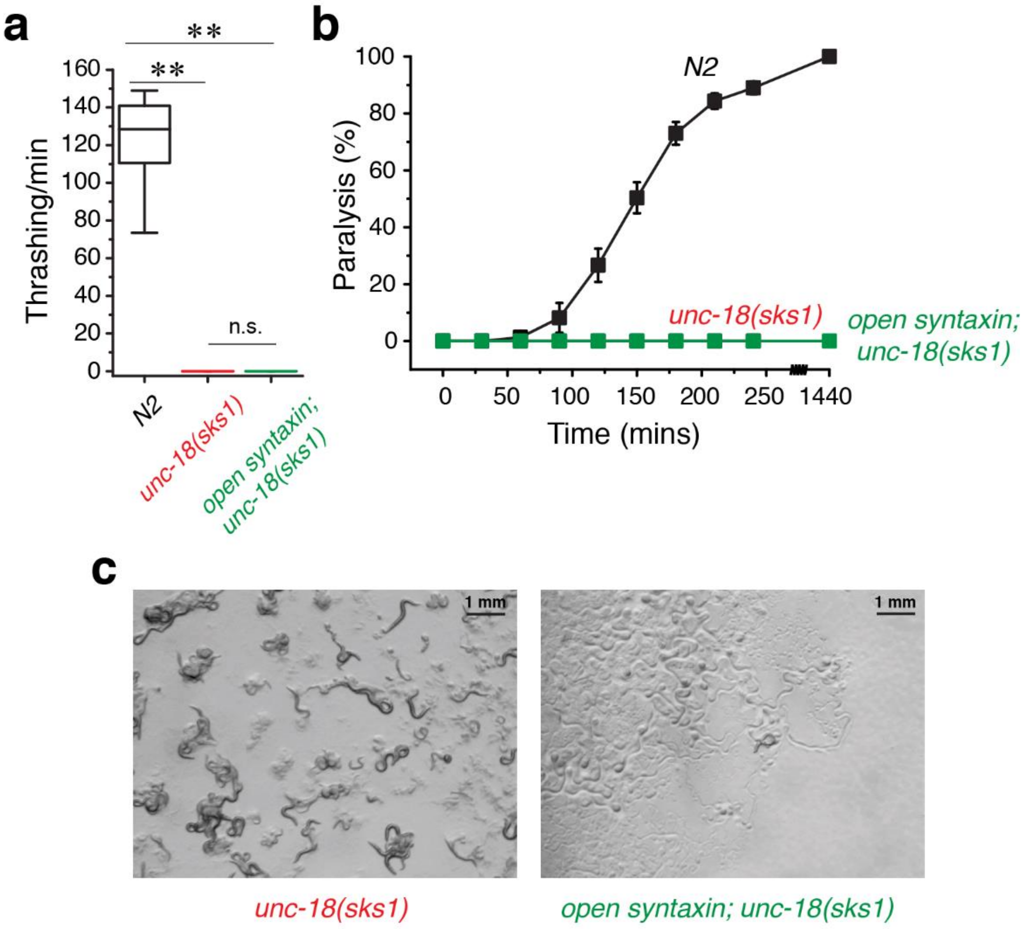

Supplementary Figure S6. The knock-in open syntaxin mutation further impairs phenotypes of *unc-18(sks1)* mutants. (a) Box-and-whisker plots of thrashing assay of N2, *unc-18(sks1)*, and *open syntaxin; unc-18(sks1)* double mutants in M9 buffer. *unc-18(sks1)* worms displayed greatly reduced thrashing rates (0.175 thrashes/min) which was reduced further by the introduction of open syntaxin in the double mutant to 0.0375 thrashes/min. n=40 for each strain. In one-way ANOVA statistical tests, $F_{(2,117)} = 607$ and $p = 0.00$. Tukey's test was performed for means analysis in ANOVA. For N2 vs *unc-18(sks1)*: ** $p = 0.00$; for *unc-18(sks1)* vs. *open syntaxin; unc-18(sks1)*: n.s. $p = 1.00$; for N2 vs. *open syntaxin; unc-18(sks1)*: ** $p = 0.00$. Box-and-whisker plots represent the median (central line), 25th–75th percentile (bounds of the box) and 5th–95th percentile (whiskers). (b) Aldicarb assays of N2, *unc-18(sks1)*, and *open syntaxin; unc-18(sks1)* double mutants. *unc-18* and *open syntaxin; unc-18* animals displayed similar resistance to aldicarb even after 24 hours. n=6 for 4-hr assays. Each assay was conducted with 15-20 worms. Error bars represent SEM. (c) Growth rate/body size images taken at day 9.

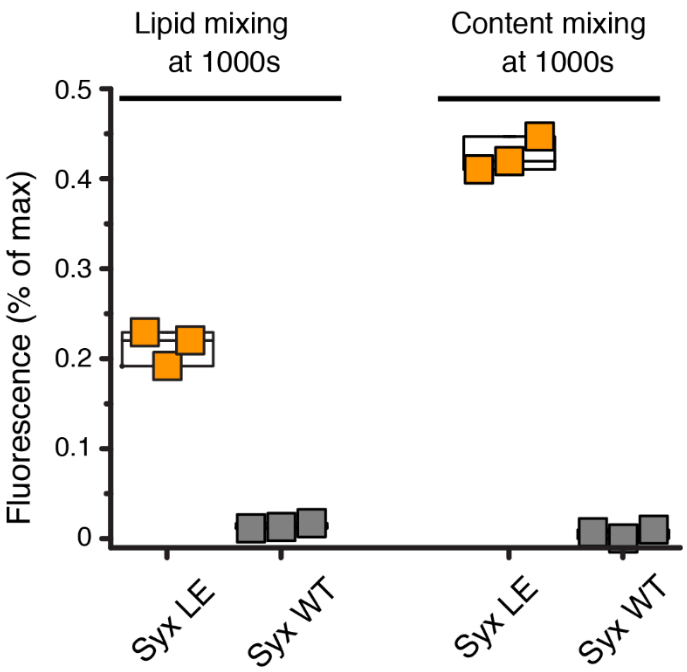

Supplementary Figure S7. Quantification of the fusion assays shown in Fig. 8c, 8d. Box-and-whisker plots overlaid with data points of normalized fluorescence intensities observed in lipid mixing (a) and content mixing (b) assays at 1,000 s, performed in triplicates. Box-and-whisker plots represent the median (central line) and 25th–75th percentile (bounds of the box).

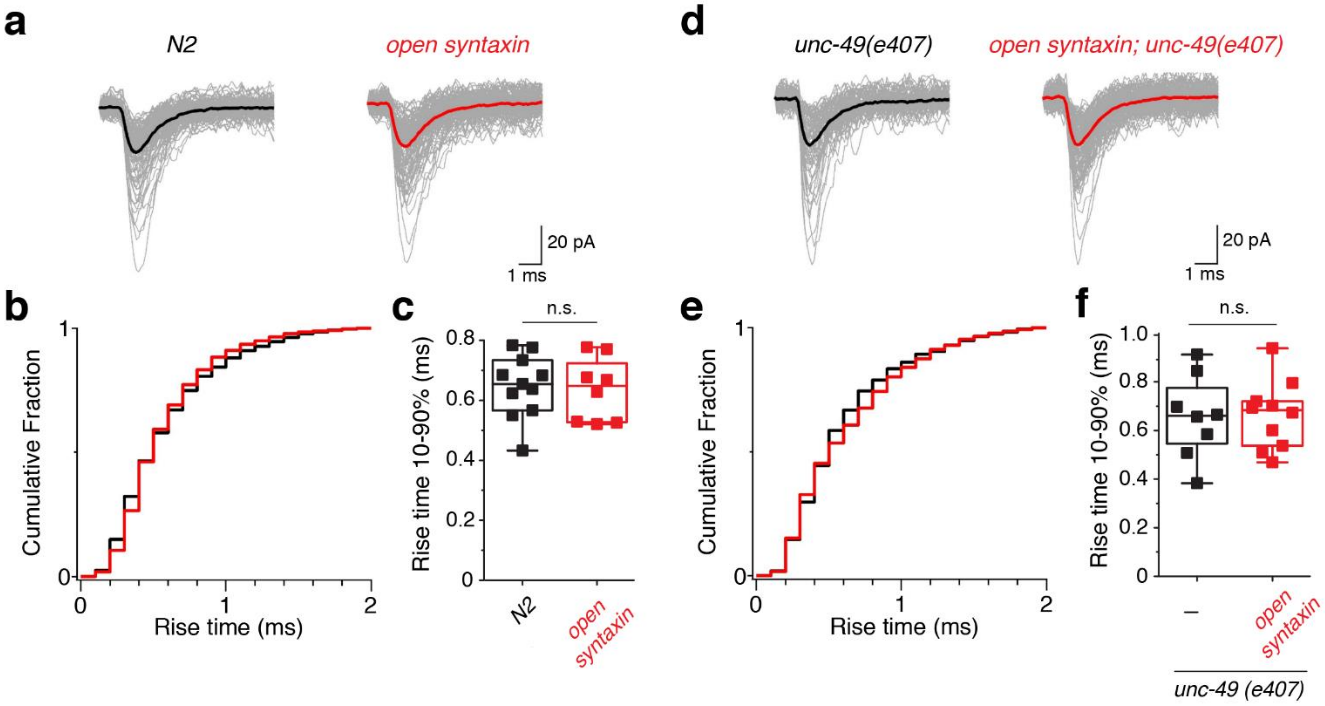

Supplementary Figure S8. The open syntaxin knock-in mutation does not affect synaptic vesicle fusion pore kinetics appreciably. (a) Representative traces of mPSCs recorded from N2 and open syntaxin KI worms. (b) Cumulative distributions of mPSC rise times in N2 wild-type (black) and open syntaxin KI (red) worms. (c) Box-and-whisker plots overlaid with data points of 10-90% rise times in (b). Two-sample two-sided t-test, n.s. $p = 0.83$. n=11 for N2 and n=8 for *open syntaxin* animals. (d). Representative traces of mEPSCs recorded from *unc-49(e407)* and *open syntaxin; unc-49(e407)* double mutant worms. (e) Cumulative distributions of mEPSC rise times in *unc-49* (black) and *open syntaxin; unc-49* (red) worms. (f) Box-and-whisker plots overlaid with data points of 10-90% rise times in (e). n.s. Two-sample two-sided t-test, n.s. $p = 0.94$. n=9 for *unc-49(e407)* and *open syntaxin; unc-49(e407)* animals. Box-and-whisker plots all represent the median (central line), 25th–75th percentile (bounds of the box) and 5th–95th percentile (whiskers).
