# Supplementary material for: Open syntaxin overcomes exocytosis defects of diverse mutants in C. elegans
Source: Nat Commun. 2020 Nov 2;11:5516. doi: 10.1038/s41467-020-19178-x (PMC7606450; doi:10.1038/s41467-020-19178-x)
Supplement: Supplementary file 3 — Reporting Summary [file 41467_2020_19178_MOESM3_ESM.pdf]

nature research

Corresponding author(s): Jose Rizo, Shangbang Gao, Shuzo Sugita

Last updated by author(s): Sep 20, 2020

Reporting Summary

Nature Research wishes to improve the reproducibility of the work that we publish. This form provides structure for consistency and transparency in reporting. For further information on Nature Research policies, see our Editorial Policies and the Editorial Policy Checklist.

Statistics

For all statistical analyses, confirm that the following items are present in the figure legend, table legend, main text, or Methods section.

n/a	Confirmed	
☐	☒	The exact sample size (*n*) for each experimental group/condition, given as a discrete number and unit of measurement
☐	☒	A statement on whether measurements were taken from distinct samples or whether the same sample was measured repeatedly
☐	☒	The statistical test(s) used AND whether they are one- or two-sided<br>*Only common tests should be described solely by name; describe more complex techniques in the Methods section.*
☐	☒	A description of all covariates tested
☐	☒	A description of any assumptions or corrections, such as tests of normality and adjustment for multiple comparisons
☐	☒	A full description of the statistical parameters including central tendency (e.g. means) or other basic estimates (e.g. regression coefficient) AND variation (e.g. standard deviation) or associated estimates of uncertainty (e.g. confidence intervals)
☐	☒	For null hypothesis testing, the test statistic (e.g. *F*, *t*, *r*) with confidence intervals, effect sizes, degrees of freedom and *P* value noted<br>*Give P values as exact values whenever suitable.*
☒	☐	For Bayesian analysis, information on the choice of priors and Markov chain Monte Carlo settings
☒	☐	For hierarchical and complex designs, identification of the appropriate level for tests and full reporting of outcomes
☒	☐	Estimates of effect sizes (e.g. Cohen's *d*, Pearson's *r*), indicating how they were calculated

Our web collection on statistics for biologists contains articles on many of the points above.

Software and code

Policy information about availability of computer code

Data collection	We used OMAX ToupView software (version 3.7) to acquire thrashing images of worms, Image J software (version 1.53a) to quantify the Western Blot data, used PULSE program software (version 8.74) to collect the electrophysiology data.
Data analysis	We used OriginPro 2016, Igor Pro (version 6.21) and Clampfit (version 10.2) softwares for data analysis.

For manuscripts utilizing custom algorithms or software that are central to the research but not yet described in published literature, software must be made available to editors and reviewers. We strongly encourage code deposition in a community repository (e.g. GitHub). See the Nature Research guidelines for submitting code & software for further information.

Data

Policy information about availability of data

All manuscripts must include a data availability statement. This statement should provide the following information, where applicable:

- Accession codes, unique identifiers, or web links for publicly available datasets
- A list of figures that have associated raw data
- A description of any restrictions on data availability

Our availability of data includes "A list of figures that have associated raw data are Figures 1a-b, 1d, 2a-b, 2d, 3b-e, 4a-b, 4d, 5a-h, 5j, 5l, 6a-f, 7a-b, 7d, 8, 9a-b, 9d, Supplementary Figures S1b-d, S3, S4,S5a-b, S6a-b, S7,S8c, S8f. " This is stated in our text too.

Field-specific reporting

Please select the one below that is the best fit for your research. If you are not sure, read the appropriate sections before making your selection.

- [x] Life sciences
- [ ] Behavioural & social sciences
- [ ] Ecological, evolutionary & environmental sciences

For a reference copy of the document with all sections, see nature.com/documents/nr-reporting-summary-flat.pdf

Life sciences study design

All studies must disclose on these points even when the disclosure is negative.

Sample size	We did not perform sample-size test. Our sample size for motility assays (thrashing) was 40 or more, because we noted a relatively large variation of behavior and because we aimed to minimize a human error in manual counting of the video-recorded thrashing. Our sample size for aldicarb assays was based on 15-20 worms for each group per assay and this assay was repeated 6 or 7 times. Our sample size for electrophysiology is 5 or larger, which we find usually sufficient to test the significance, because the electrophysiological data from the same genetic strain of worms have a small variation. This is evident in our Box-and-whisker graphs which was overlaid with the corresponding data points.
Data exclusions	No data were excluded.
Replication	We replicated the data 3-7 times and confirmed the reproducibility.
Randomization	We analyzed different genetic mutants in the same way and compared them side-by-side. Since we did NOT allocate animals in different treatment groups (e.g., treatment vs non-treatment groups), randomization is not relevant to this study.
Blinding	Behavioral (thrashing and aldicarb sensitivity) assays were performed in blind conditions because these assays involve somewhat subjective human judgments. We did not do the electrophysiology experiments in blind, because here we objectively recorded electrophysiological data from different genetic strains using the PULSE program software. The recorded parameters includes frequency and amplitude of miniature PSC and amplitude of EPSC. The analysis of these parameters is based on well established Igor Pro 6.21 and Clampfit 10.2 softwares.

Reporting for specific materials, systems and methods

We require information from authors about some types of materials, experimental systems and methods used in many studies. Here, indicate whether each material, system or method listed is relevant to your study. If you are not sure if a list item applies to your research, read the appropriate section before selecting a response.

Materials & experimental systems

n/a	Involved in the study
[ ]	[x] Antibodies
[x]	[ ] Eukaryotic cell lines
[x]	[ ] Palaeontology and archaeology
[ ]	[x] Animals and other organisms
[x]	[ ] Human research participants
[x]	[ ] Clinical data
[x]	[ ] Dual use research of concern

Methods

n/a	Involved in the study
[x]	[ ] ChIP-seq
[x]	[ ] Flow cytometry
[x]	[ ] MRI-based neuroimaging

Antibodies

Antibodies used	I378 (rabbit polyclonal anti-syntaxin-1, kind gift of Thomas Sudhof (Stanford)), Commercial E7 clone (mouse monoclonal anti-beta tubulin antibody) from Developmental Studies Hybridoma Bank. E7 is the catalogue number and its lot number is not available.
Validation	I378 polyclonal antibody has been validated extensively for detection of mammalian syntaxin-1 in the publications from Thomas Sudhof's lab. The cross reactivity with C. elegans syntaxin UNC-64 was demonstrated by our publication of Park et al. (2017, J. Neurosci.). Reactivity of E7 clone towards C. elegans beta tubulin is confirmed on their website of https://dshb.biology.uiowa.edu/E7_2.

Animals and other organisms

Policy information about studies involving animals; ARRIVE guidelines recommended for reporting animal research

Laboratory animals	C. elegans, Young adult stage of hermaphrodites were used for motility assays, aldicarb assays and electrophysiology. Growth speed was assessed by extracting eggs on the plate and taking photos of the plates daily for up to 7 days for N2 worms, and up to 9 days for the other strains.

Wild animals	The study did not involve wild animals.
Field-collected samples	The study did not involve samples collected from the field.
Ethics oversight	No ethical approval or guidance was required because the animal used in this study was nematode worms.

Note that full information on the approval of the study protocol must also be provided in the manuscript.
